# Collagen hydrogel viscoelasticity regulates MSC chondrogenesis in a ROCK-dependent manner

**DOI:** 10.1126/sciadv.ade9497

**Published:** 2023-02-10

**Authors:** Danyang Huang, Yuehong Li, Zihan Ma, Hai Lin, Xiangdong Zhu, Yun Xiao, Xingdong Zhang

**Affiliations:** National Engineering Research Center for Biomaterials, Sichuan University, Chengdu 610064, China.

## Abstract

Mesenchymal stem cell (MSC) chondrogenesis in three-dimensional (3D) culture involves dynamic changes in cytoskeleton architecture during mesenchymal condensation before morphogenesis. However, the mechanism linking dynamic mechanical properties of matrix to cytoskeletal changes during chondrogenesis remains unclear. Here, we investigated how viscoelasticity, a time-dependent mechanical property of collagen hydrogel, coordinates MSC cytoskeleton changes at different stages of chondrogenesis. The viscoelasticity of collagen hydrogel was modulated by controlling the gelling process without chemical cross-linking. In slower-relaxing hydrogels, although a disordered cortical actin promoted early chondrogenic differentiation, persistent myosin hyperactivation resulted in Rho-associated kinase (ROCK)–dependent apoptosis. Meanwhile, faster-relaxing hydrogels promoted cell-matrix interactions and eventually facilitated long-term chondrogenesis with mitigated myosin hyperactivation and cell apoptosis, similar to the effect of ROCK inhibitors. The current work not only reveals how matrix viscoelasticity coordinates MSC chondrogenesis and survival in a ROCK-dependent manner but also highlights viscoelasticity as a design parameter for biomaterials for chondrogenic 3D culture.

## Teaser

Faster-relaxing collagen hydrogel facilitates long-term chondrogenesis by mitigating myosin hyperactivation.

## INTRODUCTION

Mesenchymal stem cells (MSCs) are commonly used for tissue engineering and regenerative medicine owing to their easy accessibility and multilineage differentiation potential (*1*–*3*). Culturing MSCs in vitro with precisely orchestrated soluble agents and engineered matrices has unraveled important insights into tissue morphogenesis and homeostasis (*4*). Distinct from direct cell expansion in osteogenesis and adipogenesis, MSC chondrogenesis undergoes mesenchymal condensation, where cells exhibit extensive contraction and establish N-cadherin–mediated cell-cell adhesions that initiate chondrogenic differentiation (*5*–*7*). The contraction is a prerequisite for MSC chondrogenesis and is better supported in three-dimensional (3D) permissive matrix compared with 2D substrates (*1*, *8*, *9*). On the other hand, chondrocyte lineage-committed cells exhibit rapid phenotype drift in 2D monolayer culture, which can be redifferentiated after 3D culture recovery (*10*, *11*). Therefore, 3D culture has been considered as a favorite way for MSC chondrogenesis in vitro.

When MSCs are dissociated from monolayer, they are removed from the restraint of their substrate and cell contacts, resulting in rapid actomyosin contraction and cytoskeleton rearrangement (*12*). The dynamic changes in cell morphology are driven by Rho-associated kinase (ROCK)–dependent actomyosin contraction and modulate how MSCs integrate the mechanical and molecular information from the microenvironment (*13*). Regulation of cytoskeletal architecture is crucial for MSC lineage commitment, and the ROCK pathway has shown a critical regulatory role in MSC commitment toward adipocyte, osteoblast, or myogenic fate (*14*, *15*). It is also critical for chondrogenesis initiation, as disrupting actomyosin contraction alone is sufficient to prevent mesenchymal condensation and inhibit chondrogenic differentiation (*13*). Notably, MSC chondrogenic fate specification and morphogenesis are precisely modulated by the dynamic cytoskeleton changes, which are regulated by the interactions with the surrounding microenvironment that they inevitably pertain to.

During native cartilage development, the extracellular matrix (ECM) undergoes substantial remodeling along with the progression of chondrogenesis (*6*). Native chondrogenic differentiation is initiated after the enzymatic degradation of hyaluronan, and a type I collagen–rich ECM further facilitates mesenchymal condensation (*5*). In our previous studies, type I collagen hydrogel was able to induce MSC chondrogenic differentiation without soluble growth factors and holds translational potential in regenerating cartilage defects (*16*–*18*). The chondro-inducing effect of collagen hydrogel depends on its elastic modulus, fiber structure, and surface charge (*19*–*22*). As one of the most well-applied natural fibrillar hydrogels in cell culture and tissue engineering, collagen hydrogel exhibits distinct viscoelastic behaviors (*23*, *24*), a time-dependent mechanical property that has been recently recognized as an important regulator of the dynamic cell-matrix interactions (*25*–*27*). However, it remains elusive how this dynamic property of collagen hydrogel facilitates the cytoskeleton rearrangement of MSCs during chondrogenic lineage commitment and morphogenesis.

In this study, we examined how collagen hydrogel viscoelasticity dynamically regulates the chondrogenic differentiation at different stages of chondrogenesis. The viscoelasticity of collagen hydrogel was modulated by controlling the gelling process without adding external cross-linkers or changing collagen concentration. The regulatory role of collagen viscoelasticity on both early initiation and long-term commitment of MSC chondrogenesis was assessed via cartilage-specific gene expression and matrix deposition. Pharmacological inhibition of cytoskeletal contraction was conducted to determine the roles of viscoelasticity of collagen hydrogels in ROCK-dependent apoptosis and chondrogenesis. Thus, our study reveals an unexpected role of matrix viscoelasticity in dynamically mediating ROCK-dependent actomyosin contraction and apoptosis during MSC chondrogenesis and provides an important reference for tuning collagen hydrogel for stem cell studies.

## RESULTS

### Collagen hydrogels with various self-assembly degrees exhibit different viscoelastic behaviors

**Fig. 1.**
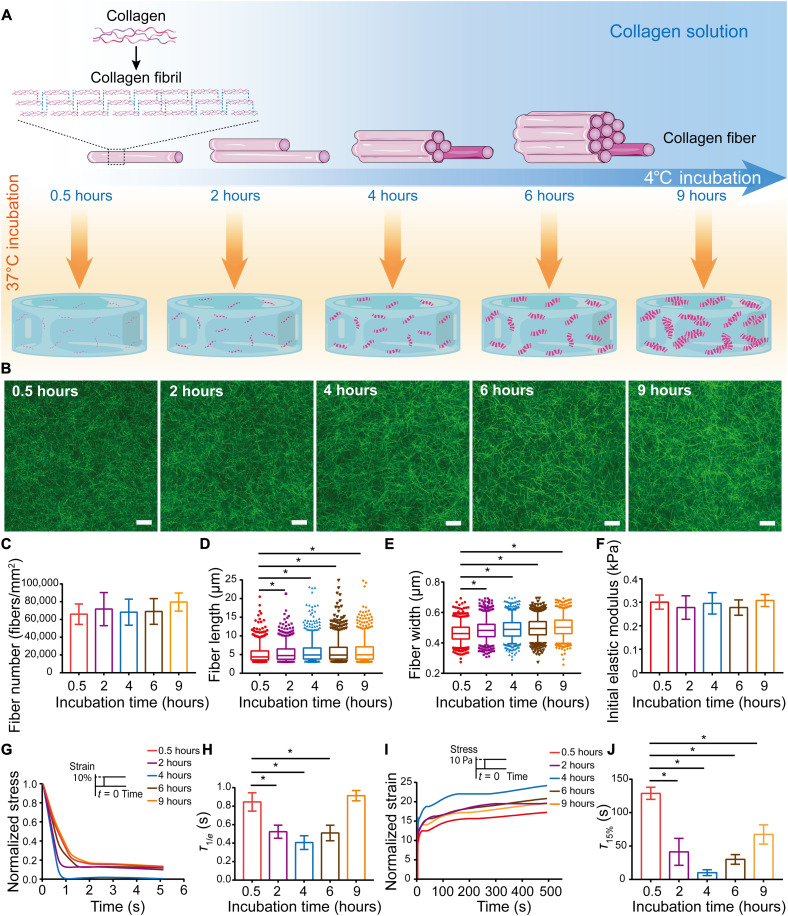
Adjusting self-assembly degree of collagen hydrogels modulates viscoelastic properties independent of initial elastic modulus. (**A**) Schematic showing the formation of hydrogels from collagen fibers with different self-assembly degrees under various low-temperature incubation times. Drawing not to scale. (**B**) Confocal microscopy images of hydrated collagen hydrogels. Scale bars, 10 μm. (**C**) Number of collagen fibers (*n* = 3; *P* = 0.7938; data are shown as means ± SD). (**D** and **E**) Length (D) and width (E) of individual collagen fibers (*n* = 3; *P < 0.05). (**F**) Initial elastic modulus as determined by compression testing of collagen hydrogels (*P* = 0.7195). Data are shown as means ± SD; one representative experiment is shown; three independent experiments; *n* = 4. (**G**) Representative stress relaxation curves of collagen hydrogels at a strain of 10%, which was normalized to the initial stress. (**H**) Time scale of stress relaxation, τ_1/*e*_, for different collagen hydrogels. *P < 0.05. Data are shown as means ± SD; one representative experiment is shown; three independent experiments; *n* = 5. (**I**) Representative creep curves of collagen hydrogels at a stress of 10 Pa, which was normalized to the initial strain. (**J**) Time scale of creep response, τ_15%_, for the different collagen hydrogels. *P < 0.05. Data are shown as means ± SD; one representative experiment is shown; three independent experiments; *n* = 3.

Collagen hydrogel has been a popular choice for stem cell and organoid culture, and the incubation conditions applied in collagen gelation directly regulate collagen fiber self-assembly in vitro (*28*, *29*). Here, we modulated collagen fiber self-assembly by adjusting the low-temperature incubation time of collagen solution (Fig. 1A). We observed an overall monotonic increase of collagen solution turbidity with prolonged incubation on ice (fig. S1), corresponding to a more extensive fibril self-assembly. During 37°C gelation, the turbidity of collagen solution with 4 hours of low-temperature incubation was prominently higher than that with 0.5 hours at equilibrium, where the self-assembly of collagen fibrils reached the plateau and the collagen fibers entangle to form a 3D network (fig. S2). The collagen solution with extended low-temperature incubation time eventually results in collagen fiber network with higher self-assembly degree. In addition, the size distribution of collagen fibers in collagen solutions after different low-temperature incubation was characterized via dynamic light scanning, and the hydrodynamic diameters of collagen fibers increased gradually with the extension of low-temperature incubation time (fig. S3). We then characterized the architecture of collagen fibers corresponding to various low-temperature incubation times. To characterize the collagen fiber architecture in situ, collagen hydrogels were examined at 488 nm in reflection mode under a confocal laser scanning microscope (CLSM). With the extended low-temperature incubation time, the collagen fiber networks were more organized and interconnected (Fig. 1B). Image analysis showed no significant difference in the total number of collagen fibers among different hydrogels (Fig. 1C), indicating an identical total number of nucleation sites. However, further quantification of individual fibers showed significant increase in both length (Fig. 1D) and width (Fig. 1E) of collagen fibers with extended low-temperature incubation. To further confirm the degree of collagen self-assembly, the microstructure of collagen fibers was characterized using scanning electron microscopy (SEM) (fig. S4A). With prolonged low-temperature incubation, the diameter of collagen fibers gradually increased, corresponding to increased collagen fiber self-assembly (fig. S4B). Atomic force microscopy (AFM) was also applied to characterize the collagen fibers (fig. S5A) and demonstrated that collagen fibers became thicker with the prolongation of incubation time at low temperature (fig. S5B). Together, these results showed that prolonging low-temperature incubation time facilitates the self-assembly of collagen fibers.

Collagen molecules self-assemble into fibrillar hydrogel at physiological temperature, and the fibrillar network is held together by electrostatic and hydrophobic bonds (*30*). Collagen hydrogel exhibits viscoelastic behaviors as these weak interactions unbind under stress, allowing for fiber slippage (*23*, *24*). Here, collagen concentrations were held constant so that their initial elastic moduli were identical (Fig. 1F). With the extended low-temperature incubation, more weak interactions were established within the collagen fiber network and resulted in different stress relaxation behaviors of the hydrogels (Fig. 1G). With the extended low-temperature incubation, the stress relaxation time (τ_1/*e*_) of collagen hydrogels decreased first and then increased, and the quickest stress relaxation was correlated to 4 hours of incubation (Fig. 1H). Creep tests were then conducted as a complement to the stress relaxation tests (Fig. 1I), and the creep time (τ_15%_) exhibited a decrease and then an increase similar to stress relaxation time (Fig. 1J). To confirm the repeatability of collagen viscoelasticity, repeated stress relaxation tests of collagen hydrogels were conducted on a rheometer (fig. S6, A to E). The initial shear modulus of each repeated test remained unchanged, indicating no damage on collagen network structure after repeated tests (fig. S6F). However, the stress relaxation time increased at every repeat, while the trend of stress relaxation time with different incubation times remained valid, where the relaxation time τ_1/*e*_ with 4 hours of incubation was the shortest (fig. S6G). Together, a series of collagen hydrogels exhibiting different viscoelastic behaviors independent of initial elastic modulus were prepared by controlling the low-temperature incubation time without any chemical cross-linking. To investigate the effect of collagen viscoelasticity on MSC chondrogenesis, collagen hydrogels with 0.5 or 4 hours of low-temperature incubation, corresponding to slower- and faster-relaxing hydrogels, were selected for the following experiments.

### Matrix viscoelasticity regulates cell morphology and actin organization

**Fig. 2.**
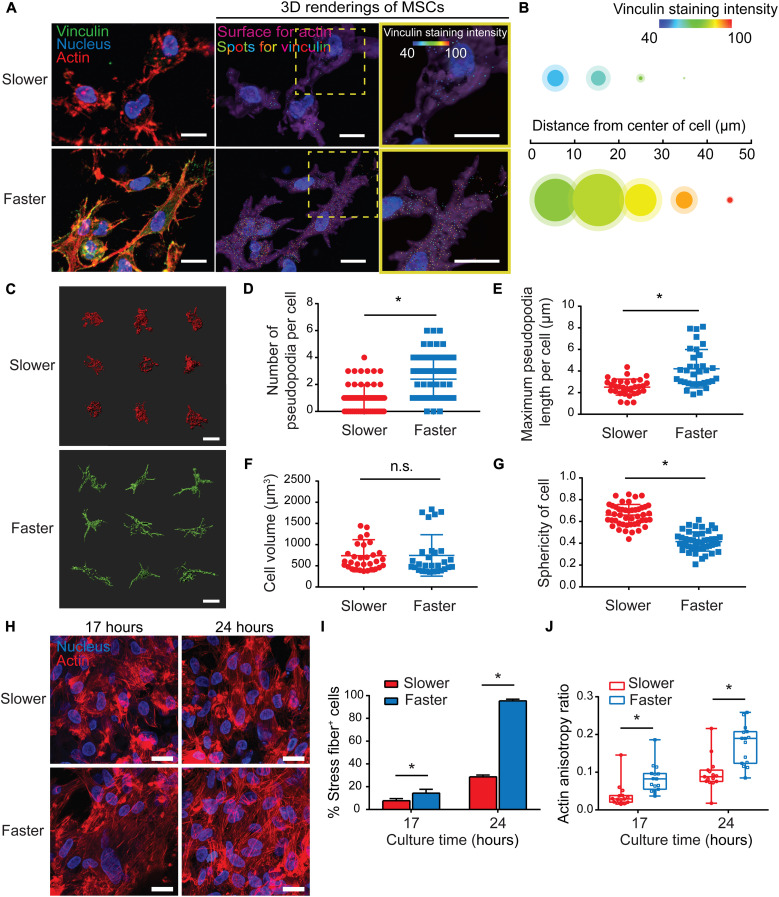
Matrix viscoelasticity modulates cell morphology and actin organization. (**A**) Vinculin immunostaining of MSCs cultured in hydrogels (3 hours). Scale bars, 10 μm. Middle images show rendered images with cytoskeleton surface in purple, nucleus in blue, and vinculin-positive staining in colorful spots corresponding to a heatmap of fluorescent intensity; right images show magnified images of the boxed areas in the middle images. Scale bars, 10 μm. (**B**) A profile illustrating average signal of vinculin expressed in single cells. The area of each circle represents the number of vinculin spots in local regions emanating from cell center, and the shaded areas demonstrate 95% confidence interval; the color of each circle represents the average fluorescence intensity (*n* = 12 single cells from three biological replications). (**C**) Representative 3D renderings of MSC cytoskeletons (3 hours). Scale bars, 10 μm. (**D** and **E**) Quantification of pseudopodia number per cell (D) and maximum pseudopodia length per cell (E) (*n* = 30 single cells from three biological replications; *P < 0.05). (**F**) Quantification of cell volume (*n* = 30 single cells from three biological replications; *P* = 0.3411). n.s., not significant. (**G**) Quantification of cell sphericity (*n* = 30 single cells from three biological replications; *P < 0.05). (**H**) Representative images of F-actin staining (17 and 24 hours). Scale bars, 20 μm. (**I**) Quantification of percentage of cells forming stress fibers (*n* = 3, *P < 0.05; data are shown as means ± SD). (**J**) Actin organization and polarization were quantified via anisotropy ratios (*n* = 15 single cells from three biological replications; *P < 0.05).

To investigate the effect of matrix viscoelasticity on stem cells in 3D culture, MSCs were encapsulated in slower- and faster-relaxing collagen hydrogels. It has been accepted that cells perceive and respond to the mechanical microenvironment via focal adhesions, and it is dictated by the matrix mechanical properties (*31*). Immunostaining against vinculin was performed as the hallmark of focal adhesions. Vinculin-rich focal adhesions were prominent at the tips of cell protrusions in the faster-relaxing group, while cells encapsulated in slower-relaxing hydrogels exhibited smaller and scattered focal adhesions distributed all over the cell membrane (Fig. 2A). Spot rendering was applied to characterize the intracellular localization and fluorescence intensity of vinculin. Intracellular localization was quantified as several vinculin spots localized within cells, and fluorescence intensity was quantified as a heatmap (Fig. 2A). Spot rendering profile quantification revealed more and stronger vinculin staining in local regions emanating from cell center in faster-relaxing hydrogels compared with that in the corresponding regions of cells in slower-relaxing hydrogels, indicating more mature focal adhesions in faster-relaxing collagen hydrogels (Fig. 2B). Adhesion-mediated signaling at the leading edge drives cell morphology change and pseudopodia formation to explore cell surroundings (*32*). Single-cell rendering of MSCs in the 3D culture showed distinct cell morphologies between slower- and faster-relaxing matrix (Fig. 2C). Quantification of pseudopodia showed that cells in faster-relaxing hydrogels exhibited more and longer pseudopodia than those in slower-relaxing hydrogels (Fig. 2, D and E). Although quantitative analysis found no significant differences in cell volume (Fig. 2F), cellular sphericity was significantly lower in faster-relaxing hydrogels (Fig. 2G), where cells exhibited stellate morphology with many slender protrusions, while those in slower-relaxing hydrogels adopted rounded morphology with a few short protrusions. These results highlighted the regulatory role of hydrogel stress relaxation on cell morphology and spreading via pseudopodia.

We then investigated whether matrix viscoelasticity regulated actin organization of MSCs. We examined how actin cytoskeleton adapts to the viscoelastic matrix after 17 and 24 hours of chondrogenic culture. Confocal characterization of actin cytoskeleton showed more potent actin stress fibers in faster-relaxing hydrogels, while the actin staining was more granule-like in slower-relaxing hydrogels (Fig. 2H). There was a significantly higher percentage of cells exhibiting stress fibers in faster-relaxing hydrogels (Fig. 2I). In addition, quantification of actin anisotropy ratio showed that the stress fibers in faster-relaxing hydrogels were more aligned, suggesting more fibrillar actin (Fig. 2J). These results suggested that faster-relaxing matrix promotes actin polymerization and intracellular mechanical transmission.

### Slower-relaxing matrix promotes the initiation but not long-term commitment of chondrogenesis

**Fig. 3.**
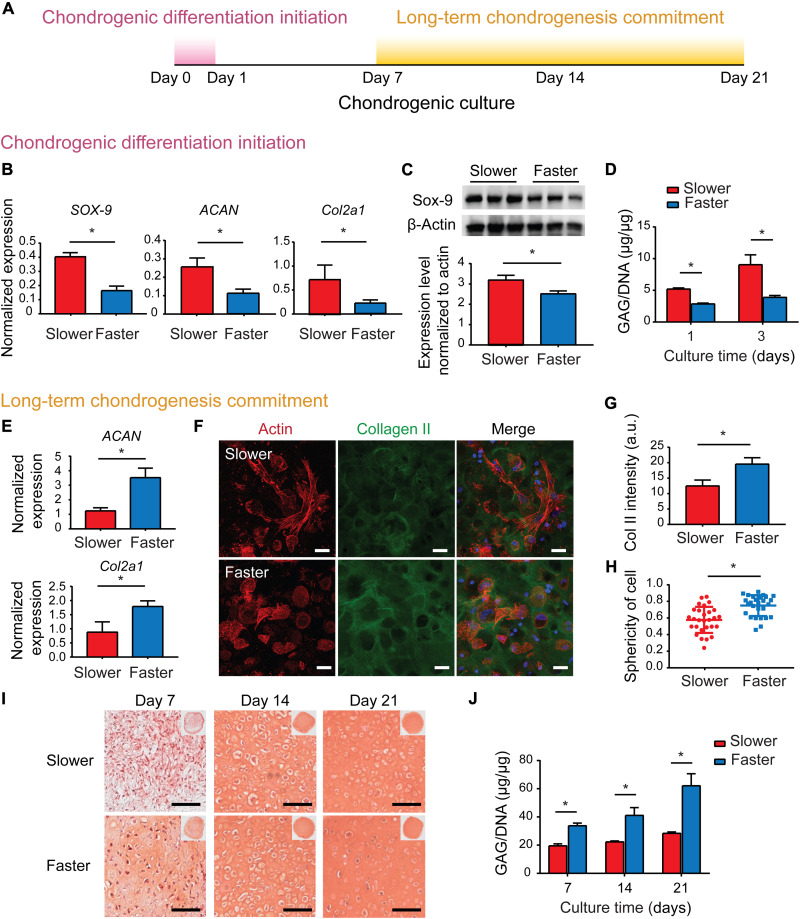
Matrix viscoelasticity plays different roles in the periods of chondrogenic differentiation initiation and long-term chondrogenesis. (**A**) Schematic of protocol to characterize chondrogenic results in different stages. (**B**) Gene expression of *SOX-9*, *ACAN*, and *Col2a1* (day 1; two independent experiments were performed, one representative experiment is shown, *n* = 4; *P < 0.05; data are shown as means ± SD). (**C**) Western blot of expression levels of Sox-9 and β-actin (day 1). Quantification of immunoblotting for Sox-9 (two independent experiments were performed, one representative experiment is shown, *n* = 3; *P < 0.05; data are shown as means ± SD). (**D**) Quantification of GAGs produced by MSCs (days 1 and 3; two independent experiments were performed; one representative experiment is shown; *n* = 3; *P < 0.05; data are shown as means ± SD). (**E**) Gene expression of *ACAN* and *Col2a1* (day 7; two independent experiments were performed; one representative experiment is shown; *n* = 4; *P < 0.05; data are shown as means ± SD). (**F**) Representative images of type II collagen immunofluorescent staining (day 7). Scale bars, 25 μm. (**G**) Quantification of type II collagen fluorescence intensity (*n* = 3, *P < 0.05; data are shown as means ± SD). a.u., arbitrary units. (**H**) Quantification of sphericity of cells (*n* = 30 single cells from three biological replications; *P < 0.05). (**I**) Representative images of Safranin O (SO) staining. Scale bars, 100 μm. (**J**) Quantification of GAGs produced by MSCs (two independent experiments were performed; one representative experiment is shown; *n* = 3; *P < 0.05; data are shown as means ± SD).

Given that spherical cell morphology facilitates MSC chondrogenic differentiation initiation, and depolymerized actin cytoskeleton promotes a series of chondrogenic regulators including sex determining region Y-box 9 (Sox-9) (*11*, *33*), we speculated that the cortical actin in slower-relaxing hydrogels facilitates chondrogenic differentiation initiation. MSCs were encapsulated in the slower- and faster-relaxing collagen hydrogels and cultured in chondrogenic medium (Fig. 3A). MSC chondrogenic differentiation initiation was promoted in slower-relaxing hydrogels indicated by up-regulated expressions of *SOX-9*, *ACAN*, and *Col2a1* on day 1 (Fig. 3B). Western blotting results confirmed higher accumulation levels of Sox-9 in slower-relaxing hydrogels on day 1 (Fig. 3C). The amount of sulfated glycosaminoglycan (sGAG), the major component of cartilage matrix, was quantified, and MSCs encapsulated in slower-relaxing hydrogels deposited significantly more sGAG content (Fig. 3D). Considering that spherical cell morphology with cortical actin promotes MSC chondrogenic differentiation (*34*–*37*), it is suggested that slower-relaxing hydrogels promoted the expressions of chondrogenic genes and cartilage matrix deposition with inhibited actin polymerization at the initiation of chondrogenic differentiation, while faster-relaxing hydrogels suppressed early chondrogenic differentiation with up-regulated actin polymerization and alignment.

To investigate whether the early chondrogenic superiority in the slower-relaxation hydrogels could be maintained, deposition of cartilage matrix at later time points was characterized. The expression levels of anabolic genes encoding type II collagen and aggrecan were compared on day 7, and the faster-relaxation matrix significantly up-regulated *Col II* and *ACAN* gene expressions (Fig. 3E). The up-regulated deposition of cartilage matrix in faster-relaxing hydrogels was further confirmed via immunofluorescent staining against type II collagen, the main component of cartilage matrix (Fig. 3, F and G). In addition, cells in fast-relaxing hydrogels exhibited spherical chondrocyte-like morphologies (Fig. 3H). With the prolongation of chondrogenic induction culture time, the cells in both groups showed spherical chondrocyte-like morphologies on day 14 (fig. S7), yet the cartilage matrix deposited by cells in the faster group was substantially higher than that in the slower group. Safranin O (SO) staining for chondroitin sulfate, another major component of cartilage matrix, showed positive staining in most faster-relaxing samples, while few positive staining was observed in the slower group on day 7 (Fig. 3I). SO-positive staining increased notably in both groups until day 21, whereas the staining intensities were always higher in the faster-relaxing hydrogels (Fig. 3I). This was further confirmed via sGAG quantification, as a significantly higher sGAG amount was deposited in hydrogels with faster stress relaxation on days 7, 14, and 21 (Fig. 3J). Together, these results suggested that the early chondrogenic superiority induced by slower-relaxing hydrogels could not be maintained, while the faster-relaxing hydrogels promoted cartilage matrix deposition during long-term MSC chondrogenesis.

### Matrix viscoelasticity regulates MSC survival in a ROCK-dependent manner

**Fig. 4.**
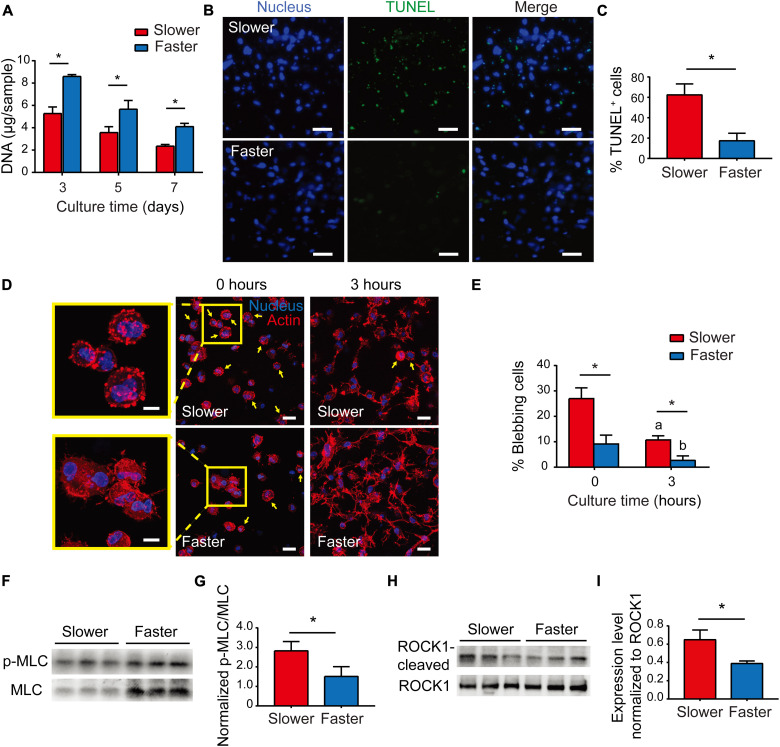
Matrix viscoelasticity modulates cell survival via regulating ROCK-dependent apoptosis. (**A**) Quantification of DNA of MSCs (two independent experiments; one representative experiment is shown; *n* = 3; *P < 0.05; data are shown as means ± SD). (**B**) Terminal deoxynucleotidyl transferase–mediated deoxyuridine triphosphate nick end labeling (TUNEL) staining of MSCs (day 3). Scale bars, 50 μm. (**C**) Quantification of TUNEL-positive cells (*n* = 5, *P < 0.05; data are shown as means ± SD). (**D**) Representative images of F-actin staining (0 and 3 hours after collagen gelation). Scale bars, 20 μm. The inserts show magnified images of blebbing cells at 0 hours. Scale bars, 10 μm. Yellow arrows point to blebbing cells. (**E**) Percentages of blebbing cells (three images from three biological replications per condition; *P < 0.05; data are shown as means ± SD); a indicates a significant difference (*P < 0.05) compared with the slower group at 0 hours; b indicates a significant difference (*P < 0.05) compared with the faster group at 0 hours. (**F**) Western blot of expression levels of p-MLC and MLC (day 1). (**G**) Quantification of immunoblotting for MLC activation (two independent experiments with one representative experiment shown; *n* = 3; *P < 0.05; data are shown as means ± SD). (**H**) Western blot of expression levels of ROCK cleaved by caspases-3 and ROCK (day 3). (**I**) Quantification of immunoblotting for cleavage of ROCK (two independent experiments; one representative experiment is shown; *n* = 3; *P < 0.05; data are shown as means ± SD).

We then explored the reason for the unsustainability of chondrogenic superiority in the slower-relaxing hydrogels. Given that high cell density promotes MSC chondrogenic differentiation (*38*–*40*), changes in MSC population were assessed via DNA quantification. The cell number decreased in chondrogenic culture, as the amount of DNA decreased in both groups over time, and the cell number in faster-relaxing hydrogels was significantly higher at every time point (Fig. 4A). Cell apoptosis was assessed via terminal deoxynucleotidyl transferase–mediated deoxyuridine triphosphate nick end labeling (TUNEL) staining, and it was more prominent in gels with slower stress relaxation on day 3 (Fig. 4, B and C). These results showed that MSCs underwent apoptosis during chondrogenic culture while the apoptosis was mitigated in faster-relaxing hydrogels. Therefore, the mitigated cell apoptosis might account for the up-regulated cartilage matrix deposition in faster-relaxing hydrogels during long-term MSC chondrogenesis.

Previous studies reported that when anchorage-dependent cells such as MSCs are suspended in solution or matrix without timely establishment of cell adhesion, they exhibit excessive actomyosin contraction, alter phenotypes, and proceed to cell blebbing (*41*–*43*). Pronounced cell blebbing is the result of hyperactive actin-myosin cytoskeletal contraction, often a prelude to ROCK-dependent apoptosis (*43*–*45*). Here, right upon gelation of collagen-MSC solutions incubated at 37°C for 0.5 hours, bleb formation was observed on MSC within both hydrogels (Fig. 4D), while there were significantly less blebbing cells in faster-relaxing hydrogels than in slower-relaxing hydrogels (Fig. 4E). Cell blebbing happened immediately upon dissociation and proceeded until the cells establish functional focal adhesions with surrounding collagen fibers, where the actomyosin contraction is balanced by the anchoring force (*43*). After 3 hours of culture, there were still blebbing cells in slower-relaxing hydrogels, while most cells in faster-relaxing hydrogels were spread out (Fig. 4, D and E). These results implicated that faster stress relaxation of the matrix suppressed cell blebbing at the beginning of the 3D hydrogel culture. Cell blebbing is the consequence of persistent contraction yielded by myosin hyperactivation, owing to an overexpression of myosin light-chain (MLC) phosphorylation through kinases, such as ROCK or MLC kinase (*43*). Western blot analysis showed that the phosphorylation of MLC (serine-19), corresponding to the myosin hyperactivation (*46*), was significantly higher in MSCs cultured in slower-relaxing hydrogels (Fig. 4, F and G). These results suggested that faster-relaxing hydrogels mitigated cell blebbing through suppressing myosin hyperactivation. As the downstream of persistent myosin hyperactivation, ROCK1 cleavage by caspase-3 was assessed via Western blot analysis as an indicator of ROCK-dependent apoptosis (Fig. 4H), and the results showed down-regulated ROCK1 cleavage of MSCs in faster-relaxing hydrogels (Fig. 4I). Together, these findings indicated that faster-relaxing hydrogels inhibited cell apoptosis through mitigating myosin hyperactivation in a ROCK-dependent manner, which might account for the up-regulated cartilage matrix deposition during long-term MSC chondrogenesis.

### Matrix viscoelasticity mediates long-term chondrogenesis through regulating MSC survival in a ROCK-dependent manner

**Fig. 5.**
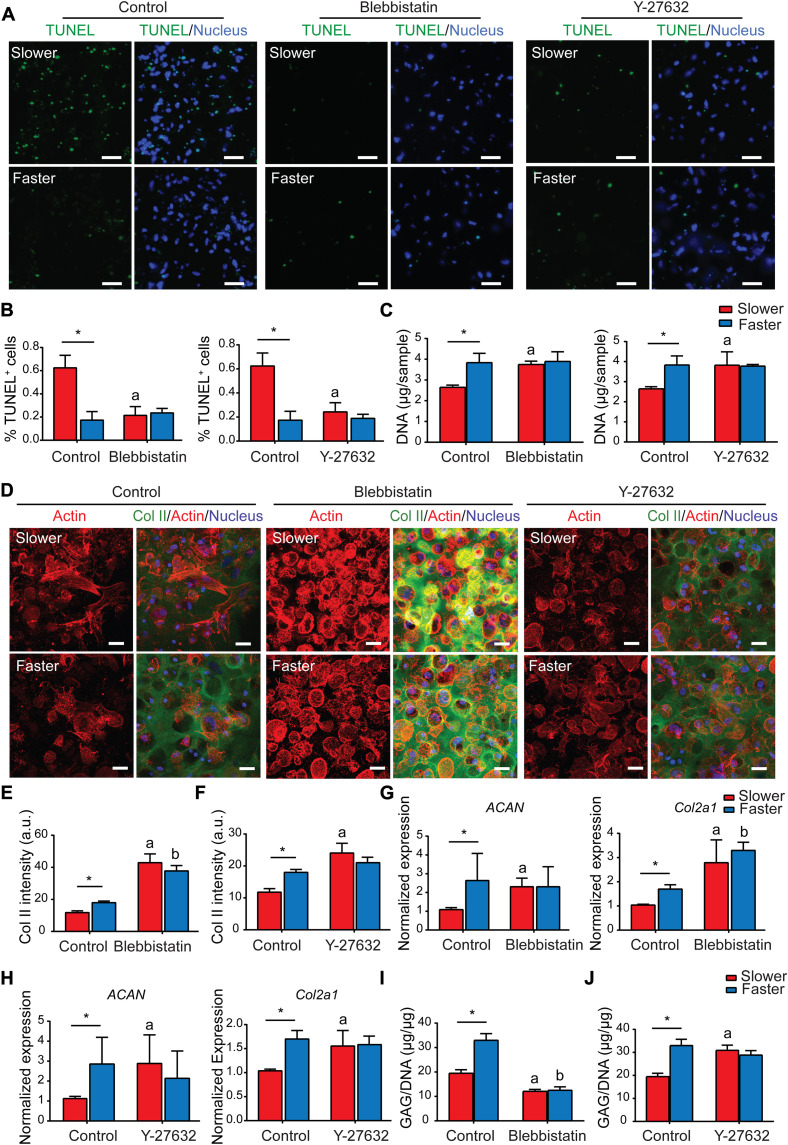
Matrix viscoelasticity mediates long-term commitment of MSC chondrogenesis through regulating cell survival via adjusting ROCK-dependent apoptosis. (**A**) TUNEL staining images of MSCs treated with blebbistatin or Y-27632 (day 2). Scale bars, 50 μm. (**B**) Quantification of TUNEL-positive cells (*n* = 3, *P < 0.05; data are shown as means ± SD). (**C**) Quantification of DNA of MSCs treated with blebbistatin or Y-27632 (day 6; two independent experiments; one representative experiment is shown; *n* = 3; *P < 0.05; data are shown as means ± SD). (**D**) Representative images of type II collagen immunofluorescent staining of MSCs treated with blebbistatin or Y-27632 (day 6). Scale bars, 25 μm. (**E** and **F**) Quantification of type II collagen fluorescence intensity of MSCs treated with blebbistatin (E) or Y-27632 (F) (*n* = 3; *P < 0.05; data are shown as means ± SD). (**G** and **H**) Gene expression of *ACAN* and *Col2a1* of MSCs treated with blebbistatin (G) or Y-27632 (H) (day 6; three independent experiments; one representative experiment is shown; *n* = 3; *P < 0.05; data are shown as means ± SD). (**I** and **J**) Quantification of GAGs produced by MSCs treated with blebbistatin (I) or Y-27632 (J) [day 6; two independent experiments; one representative experiment is shown; *n* = 3; *P < 0.05; a and b indicate a significant difference (*P < 0.05) compared with the slower and faster hydrogels in the control group, respectively; data are shown as means ± SD].

Our hypothesis thus far is that the unsustainability of early chondrogenic superiority in slower-relaxing hydrogels was attributed to up-regulated cell apoptosis caused by ROCK-dependent myosin hyperactivation. To verify that faster-relaxing hydrogels promote overall MSC chondrogenesis through suppressing ROCK-dependent apoptosis, cell apoptosis was characterized after pharmacological inhibition of ROCK pathways. The nontreated control groups exhibited significantly higher cell apoptosis in slower-relaxing hydrogels as observed above (Fig. 5, A and B). In contrast, inhibition of ROCK with Y-27632 and nonmuscle myosin II with blebbistatin abolished the difference in cell apoptosis on day 3 (Fig. 5, A and B). In addition, DNA qualification showed increased cell number in slower-relaxing hydrogels after Y-27632 and blebbistatin treatment (Fig. 5C). Therefore, the pharmacologic inhibitors mitigate MSC apoptosis caused by myosin hyperactivation similar to the effect of faster-relaxing hydrogels.

Next, cartilage matrix deposition in the two groups was then characterized after rescuing cell survival by pharmacological inhibitors. Detailed analysis of the actin cytoskeleton showed chondrocyte-like spherical cell morphology in the slower-relaxing hydrogels after blebbistatin and Y-27632 treatment, while the cells in faster-relaxing hydrogels showed no significant differences after treatment (Fig. 5D and fig. S7). Blebbistatin treatment significantly promoted type II collagen deposition visualized via type II collagen immunostaining in both groups, while Y-27632 treatment only promoted type II collagen deposition in slower-relaxing hydrogels and had no significant effect on those in faster-relaxing hydrogels (Fig. 5, D to F). Gene expression analyses revealed that the expression levels of *ACAN* and *Col2a1* were significantly up-regulated in slower-relaxing hydrogels after blebbistatin and Y-27632 treatment, while there was no significant difference in the faster-relaxation group (Fig. 5, G and H). Suppressing cell apoptosis with blebbistatin decreased the deposition of sGAG in both groups (Fig. 5I), while Y-27632 promoted sGAG deposition only in the slower-relaxing hydrogels (Fig. 5J). Together, suppressing ROCK-dependent apoptosis with blebbistatin and Y-27632 promoted overall chondrogenesis in slower-relaxing hydrogels, indicating that the unsustainability of chondrogenic superiority in slower-relaxing hydrogels was attributed to the cell apoptosis induced by ROCK-dependent myosin hyperactivation, and faster-relaxing hydrogels promoted overall chondrogenesis by mitigating ROCK-dependent apoptosis similar to the effect of pharmacological inhibition of the ROCK pathway.

## DISCUSSION

**Fig. 6.**
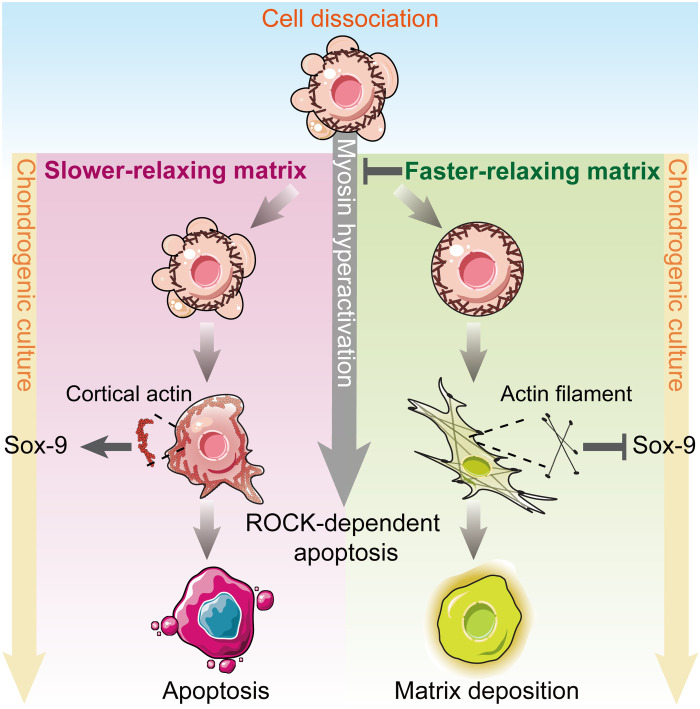
Viscoelasticity of collagen hydrogels mediates MSC chondrogenic differentiation through actin organization and cell survival via regulating ROCK-dependent actomyosin-based contractility. Schematic of the proposed mechanism explaining how viscoelasticity of collagen hydrogels regulates MSC chondrogenesis in a ROCK-dependent manner.

The regulatory role of matrix viscoelasticity on MSC chondrogenesis is not well understood despite the recently growing recognition of viscoelasticity in regulating cell expansion, proliferation, and lineage commitment. MSC chondrogenesis is unique, as the cells exhibit actomyosin contraction during mesenchymal condensation before morphogenesis, whereas cells mainly exhibit expansion forces when committing to osteoblast or adipogenic lineages. Mesenchymal condensation involves dynamic changes in cytoskeleton architecture and actomyosin contractility, which is regulated by the interaction with surrounding matrix. In this study, we show that viscoelasticity of collagen hydrogels mediates overall MSC chondrogenesis through regulating cellular chondrogenic differentiation initiation and subsequent cell survival via adjusting ROCK-dependent actomyosin-based contractility (Fig. 6). Our data demonstrate that without timely establishing interactions with surrounding matrix in slower-relaxing collagen hydrogels, MSCs exhibit persistent myosin hyperactivation. Although a disordered cortical actin architecture promotes the expression of Sox-9 at the initiation of chondrogenic differentiation, the persistent myosin hyperactivation results in ROCK-dependent apoptosis, leading to an unsustained chondrogenic superiority in slower-relaxing hydrogels. In contrast, although early chondrogenic differentiation is suppressed in faster-relaxing hydrogels with up-regulated actin polymerization, ROCK-dependent apoptosis is suppressed by mitigating myosin hyperactivation, leading to an improved long-term commitment of MSC chondrogenesis.

In this study, type I collagen was chosen as the 3D culture matrix for the following reasons: (i) type I collagen is the main ECM composite during developmental mesenchymal condensation at the initiation of chondrogenesis (*5*, *47*), (ii) collagen hydrogel induces MSC chondrogenic differentiation in vitro and holds good potential in regenerating hyaline cartilage in vivo (*8*, *48*), and (iii) collagen hydrogel exhibits distinct viscoelastic behaviors that can be tuned by controlling its gelation conditions without changing its elastic modulus. Compared with other nonfibrillar hydrogels such as hyaluronic acid, MSC-loaded collagen hydrogel exhibits excessive gel contraction during the first day of culture in our previous experiments (*18*). The contraction is attributed to cell-generated forces, which is exerted by the actomyosin cytoskeleton, transmitted across cell-ECM and cell-cell adhesions, and dissipated along the collagen fibers over long distance (*49*–*51*). Our data demonstrated important implications of collagen hydrogel viscoelasticity in coordinating MSC chondrogenesis. The degree of collagen self-assembly was adjusted by incubating collagen solution at low temperature at different times, resulting in different viscoelastic behaviors. Considering that cells bind to ECM over a time scale of ∼1 s and form stable adhesions on a time scale of minutes (*52*, *53*), 3D culture matrices were prepared with a similar stress relaxation time scale. Viscoelasticity of collagen hydrogel is attributed to the unbinding and rebinding of weak interactions between collagen fibers, such as hydrophobic and electrostatic interaction (*23*, *24*). When collagen hydrogel is subjected to external force, the unbinding of these weak bonds leads to viscoelasticity caused by the following two reasons: (i) hydrogels relax internal stress through dissipating energy by unbinding of these weak bonds (*54*) and (ii) the slippage of collagen fibers after unbinding of these weak bonds (*23*, *26*). In our study, we found quicker stress relaxation and creep behavior with increased collagen self-assembly, yet the relaxation rate is slowed down when the self-assembly degree is too high. We speculated that it is the result of number-dependent unbinding of weak bonds between collagen fibers. With the extended low-temperature incubation, more collagen fibers were assembled with more interfiber weak bonds (*55*–*57*). Under a certain degree of self-assembly, the weak bonds unbind simultaneously upon exposure to external force or deformation, and more weak bonds result in more energy dissipation and fiber slippage, which translates to overall faster stress relaxation. Nevertheless, when the collagen self-assembly degree continues to increase with the prolonged low-temperature incubation, the number of weak bonds is too large to be unbound simultaneously under the external force, resulting in the difficulty of collagen fiber slippage, thus showing an impaired stress relaxation. Note that a general 30-min incubation at 37°C without considering the preincubation time on ice is applied for current 3D culture protocols, which could be optimized more precisely as suggested by our data.

In collagen hydrogels for 3D culture, cell-generated forces are transmitted to collagen fibers and remodel the matrix, resulting in local clustering of adhesion ligands that facilitates focal adhesion formation and cell spreading along collagen fibers (*58*, *59*). In faster-relaxing hydrogels, prominent and punctate vinculin clusters, the hallmark of mature focal adhesions (*60*), were observed at protrusive tips of spreading MSCs. This is consistent with recent studies reporting that faster stress–relaxing matrix promotes the formation and maturation of focal adhesions (*61*, *62*). Moreover, cells in faster-relaxing hydrogels exhibited more and slender pseudopodia than those in slower-relaxing hydrogels. These data highlight a promoted cell-ECM interaction in faster-relaxing collagen hydrogel compared with that in slower-relaxing hydrogels. Because of quicker dissipation of the anchoring force to matrix, MSCs in faster-relaxing hydrogels exert stronger tensions to remodel surrounding matrix, thereby stabilizing the cell-ECM interaction. Consequently, the stronger cell-ECM interactions facilitated actin polymerization and stress fiber formation, resulting in a more robust cytoskeleton organization. However, it has been widely accepted that disrupted cytoskeletal organization by biophysical or biochemical stimuli promotes chondrogenic lineage commitment (*11*, *13*, *33*, *63*). Previous studies have reported that disrupting action organization promotes the expression of Sox-9, a key chondrogenic promoter, by regulating protein kinase (PK) and extracellular signal–regulated kinase (ERK)–related signal pathways, so as to up-regulate chondrogenic differentiation (*11*, *33*, *37*). Sonn and colleagues reported that disruption of actin cytoskeleton by cytochalasin D induces chondrogenesis of mesenchymal cells by activating PKC and by inhibiting ERK signaling (*37*). Subramanian and colleagues reported that disrupting actin organization by continuous low-intensity ultrasound not only promoted Sox-9 gene expression by increasing ERK1/2 phosphorylation but also enhanced the nuclei translocation of Sox-9 (*33*). In addition, Kumar and Lassar (*11*) demonstrated that actin depolymerization augments Sox-9 function by either regulating the extent of Sox-9 S181 phosphorylation by PKA or regulating the association of Sox-9 phospho-S181 with transcriptional coactivators. Here, our data indicate that the more robust cytoskeleton organization in faster-relaxing hydrogels down-regulates Sox-9 expression, while the disordered cortical actin in slower-relaxing hydrogels up-regulates Sox-9 expression. Together, it is evident that viscoelasticity of collagen hydrogels mediates the initiation of MSC chondrogenic differentiation, and faster relaxation suppresses Sox-9 expression through promoting actin polymerization and stress fiber formation.

As chondrogenic differentiation proceeds, however, the faster-relaxing hydrogels were more conducive to anabolic gene expression and cartilage matrix deposition. Lee *et al.* (*64*) demonstrated that alginate hydrogels with faster relaxation promote chondrocyte matrix deposition, while slower-relaxing hydrogels restrict cell volume expansion and led to increased secretion of interleukin-1β, which results in cartilage matrix degradation and cell death. Although we also observed that faster-relaxing hydrogels were more conducive to cartilage matrix deposition, the underlying mechanisms are different: Alginate hydrogels with different stress relaxation regulate chondrocytes’ behaviors by restricting or facilitating cell volume expansion, whereas no significant differences in cell volume were observed in collagen hydrogels in this study (fig. S8). We speculated that the difference observed here is mainly attributed to cell-matrix interactions while alginate matrix is considered bioinert. Moreover, the relatively low elastic modulus of collagen hydrogels exerts less cell volume confinement, which is insufficient to regulate cartilage matrix deposition.

Considering the remarkable decrease in cell number resulting from cell apoptosis, we speculated that lower cell density accounts for the unsustained chondrogenic superiority in slower-relaxing hydrogels. When stem cells are dissociated for 3D culture, the disruption of the junctions with ECM and adjacent cells breaks the balance between intracellular contraction and opposing anchoring forces of ECM, leading to myosin hyperactivation and cell blebbing, a predictive event of ROCK-dependent apoptosis (*41*–*43*, *65*). Here, we observed significant cell blebbing caused by myosin hyperactivation once the MSCs were encapsulated in collagen hydrogels. After cells were encapsulated in collagen hydrogels, the opposing anchoring forces on collagen fibers gradually counteract the intracellular contractive force. Faster-relaxing hydrogels promote the cell-ECM interactions, which counteracts the intracellular contractive force and reduces the excessive actomyosin tension more quickly. Consequently, ROCK-dependent apoptosis induced by myosin hyperactivation was significantly down-regulated in faster-relaxing hydrogels, suggesting a previously unreported mechanism by which collagen viscoelasticity mediates cell survival in a ROCK-dependent manner.

Y-27632 and blebbistatin were then applied as pharmacological inhibitors for ROCK pathways. Considering the critical role of actomyosin contractility in early chondrogenic lineage commitment, Y-27632 and blebbistatin treatment were not applied until a significant up-regulated Sox-9 expression after 24 hours. In line with previous studies (*42*–*44*, *66*, *67*), ROCK-dependent apoptosis in slower-relaxing hydrogels was effectively inhibited with Y-27632 or blebbistatin treatment. Therefore, our data suggest that faster-relaxing hydrogels improve MSC survival via mitigating ROCK-dependent apoptosis similar to ROCK inhibitors. The underlying mechanism of the turnover of chondrogenic superiority in slower-relaxing hydrogels involves ROCK-dependent apoptosis caused by hyperactive actomyosin contractility. As higher cell density facilitates MSC chondrogenesis with up-regulated intercellular interactions (*39*, *40*, *68*), we hypothesized that MSC chondrogenesis in faster-relaxing hydrogels is up-regulated at the cellular level. This was confirmed by the quantification of anabolic gene expression and cartilage matrix deposition. Mitigating ROCK-dependent apoptosis by Y27632 or blebbistatin treatment significantly up-regulated cartilage matrix deposition in slower-relaxing hydrogels to a level comparable to that in untreated faster-relaxing hydrogels. Together, long-term commitment of MSC chondrogenesis is mediated by collagen hydrogel viscoelasticity through regulating cell survival in a ROCK-dependent manner.

In summary, our data suggest an important role of collagen hydrogel viscoelasticity in regulating cell-matrix interactions and MSC chondrogenesis. Collagen hydrogel viscoelasticity regulates focal adhesion formation and maturation and mediates the initiation of MSC chondrogenic differentiation at early mesenchymal condensation stage via ROCK-dependent actomyosin contraction and later chondrogenesis via ROCK-dependent cell apoptosis. Overall, this study elucidates mechanistic insight into stem cell mechanotransduction during chondrogenesis and presents potential criteria for tuning collagen hydrogel as 3D matrix for cell or organoid culture in vitro. Similar criteria should be considered when designing biomaterials used in tissue engineering and regenerative medicine.

## MATERIALS AND METHODS

### Preparation of collagen hydrogels

The fabrication of collagen hydrogels was adapted from previously established protocols (*29*). Briefly, type I collagen was extracted from calf skin by dealing with pepsin (Sigma-Aldrich, USA) and solubilized in 0.02 M hydrochloric acid (Kelong Chemical Industry, China) under a sterile condition. To obtain collagen solution at the target concentration, a metric of 0.2 M NaOH was added into α-modified Eagle’s medium (α-MEM; HyClone, USA) until the color of medium turned to dark pink. Then, the high-concentration acidic collagen solution was diluted with the dark pink solution to produce a neutralized collagen solution at a final collagen concentration of 3 mg/ml. Next, the neutralization collagen solution was prepolymerized at low temperature (4°C) for various incubation periods (0.5, 2, 4, 6, and 9 hours). The resulting solution was injected into polydimethylsiloxane (PDMS) molds with 8 mm in diameter and 4 mm in height and incubated at 37°C for 30 min to construct hydrogels.

### UV measurements: Kinetics of collagen self-assembly

The kinetics of collagen self-assembly at 4°C was characterized as a lower-temperature turbidity curve immediately as soon as the neutralization of collagen solution was completed; the kinetics of collagen self-assembly at 37°C was characterized as a higher-temperature turbidity curve immediately as soon as the low-temperature incubation for various times was finished. The absorbance increase was recorded at 313 nm as a function of time. The measurements were performed by means of ultraviolet (UV)–visible spectrophotometer UR-3900 equipped with a thermostat, according to the method of Leo *et al.* (*69*).

### Digital light scattering measurements

The size distributions of collagen fibers in collagen solutions (3 mg/ml) with different low-temperature incubation times (0.5, 2, 4, 6, and 9 hours) were determined by Zetasizer (Nano ZS90, Malvern Instruments Ltd., Malvern, UK) equipped with a 633-nm laser. Collagen solution samples were transferred to a small-volume polystyrene cuvette. Backscatter measurements were performed (θ = 90° and *T* = 4°C) following a 2-min thermal equilibration. All measurements were performed in triplicate.

### Reflective imaging of collagen fibers

Collagen fiber architecture was visualized with a CLSM (Leica-TCS-SP5). Each collagen hydrogel was immersed in 1× phosphate-buffered saline (PBS) and placed on a 63× oil immersion inverted objective (Leica). Reflection microscopy was configured to capture 488-nm light reflected during illumination with the 488-nm lasers. The number of collagen fibers and the length and width of individual collagen fiber were quantified using CT-FIRE software.

### Scanning electron microscopy

The microstructures of collagen fibers were characterized using a SEM (Hitachi S-4800) operating at 5 kV. Collagen hydrogel samples were prepared as mentioned above and were rinsed in deionized water for 48 hours. The samples were then fixed with 2.5% glutaraldehyde for 40 min and dehydrated through an ascending series of ethanol concentrations up to 100%. Next, the samples were dried in a critical point dryer and gold-sputtered for imaging. Diameters of collagen fibers were quantified using DiameterJ, an ImageJ plug-in (*70*).

### Atomic force microscopy

After different low-temperature incubation times (0.5, 2, 4, 6, and 9 hours), a 10-μl sample was deposited on a freshly cleaved mica substrate and incubated at 37°C for 30 min to gel. The mica was then washed gently with deionized water to avoid salt crystallization. Last, the samples were air-dried and imaged using AFM (Dimension ICON, Bruker, Germany) with a Tapping Mode Probe tip.

### Mechanical characterization

The initial elastic modulus and stress relaxation properties of collagen hydrogels with various low-temperature incubation times were measured from unconfined compression tests of hydrogel disks (8 mm in diameter and 2 mm thick, equilibrated in PBS overnight) using a dynamic mechanical analyzer (TA-Q800, USA). The hydrogel disks were applied with a compressional deformation rate of 0.1 mm/min^−1^. The stress-versus-strain relations of the collagen hydrogels were almost linear, and the initial elastic modulus was calculated as the slope of the resulting stress/strain curves (first 5 to 10% of strain). For the stress relaxation measurements, the hydrogels were compressed to 10% strain under stress relaxation mode; consequently, the strain was held constant and then stress was recorded as a function of time. The stress relaxation time, τ_1/*e*_, was quantified as the time for which the initial stress of the hydrogel was relaxed to 1/*e* of its original value.

Shear creep tests were conducted using a stress-controlled rheometer (TA Instruments, DHR-2, USA). Once the low-temperature incubation for various times was accomplished, collagen solution was directly deposited between two plates (25 mm diameter) of the rheometer and polymerized at 37°C for 30 min. Subsequently, creep test was performed. The shear strain was recorded over time after a constant shear stress of 10 Pa was applied and the creep time, τ_15%_, was quantified as the time for the shear strain to reach 15% of its initial value immediately following the application of stress.

### Repeated stress relaxation tests

The repeated stress relaxation tests of collagen hydrogel samples were conducted on a rheometer (AntonPaar, MCR302, Austria). Once the low-temperature incubation was accomplished, collagen solution was directly deposited between two plates (25 mm diameter) of the rheometer and polymerized at 37°C for 30 min. Subsequently, three repeated stress relaxation tests were performed. The hydrogels were applied with 10% constant shear strain under stress relaxation mode with the consequent stress recorded as a function of time. There was no time interval between each test, and all the tests were in the same direction. The stress relaxation time, τ_1/*e*_, was quantified as the time for the relaxation modulus to reduce to 1/*e* of its original value.

### MSC isolation and culture

Neonatal rabbits were purchased from the Breeding Farm for Sichuan Provincial Experimental Animal Special Committee (Chengdu, China). The animal experiments were approved by the Institutional Animal Care and Use Committee of Sichuan University. MSCs were isolated from the bone marrow cavity following a standard protocol developed and validated in our group (*20*, *71*). Briefly, long bones were removed from neonatal (3- to 5-day) rabbits under aseptic conditions. Mononuclear cells were flushed from bone marrow cavities with α-MEM containing 20% fetal bovine serum (FBS; GE HealthCare, USA) and 10% penicillin/streptomycin (HyClone, USA) and maintained at 37°C in a humidified atmosphere with 5% CO_2_. After 24 hours, MSCs were selected by adherence and cultured in fresh α-MEM containing 10% FBS and 1% penicillin/streptomycin with replacement every 2 days. Passage 2 cells were harvested for further hydrogel encapsulation.

### Encapsulation of cells within hydrogels

MSCs were suspended in neutralized collagen solutions (3 mg/ml) with a low-temperature incubation time for 0.5 or 4 hours to reach a final cell density of 5 × 10^6^ cells/ml, determined using a cell counter. The mixture was then deposited in PDMS molds (Ф 8 mm by 4 mm) and gelatinized at 37°C for 30 min to construct MSCs/hydrogels. Subsequently, the constructs were cultured in high-glucose Dulbecco’s MEM (HyClone, USA), supplemented with recombinant human transforming growth factor–β1 (10 ng/ml; PeproTech, USA), 0.1 mM nonessential amino acids (Gibco, USA), 1% ITS (insulin-transferrin-sodium selenite, Sigma-Aldrich, USA), l-proline (40 mg/ml; Sigma-Aldrich, USA), 100 nM dexamethasone (Sigma-Aldrich, USA), ascorbic acid 2-phosphate (91.5 μg/ml; Sigma-Aldrich, USA), penicillin/streptomycin (100 U/ml), and 5% CO_2_ in an incubator at 37°C. Medium was regularly changed every 2 days.

### Fluorescent confocal microscopy of F-actin cytoskeleton

F-actin cytoskeleton was visualized 3 hours after gelation with a CLSM (Leica-TCS-SP5, Germany). Hydrogel constructs were prepared for fluorescent staining by first fixing MSCs with 4% paraformaldehyde for 24 hours and permeabilized with 0.5% Triton X-100 in 1× PBS for 5 min. Hydrogel constructs were then stained with Alexa Fluor 594 phalloidin (50 μg/ml; Sigma-Aldrich, USA) for F-actin cytoskeleton visualization for 40 min and 4′,6-diamidino-2-phenylindole (DAPI; Sigma-Aldrich, USA) for cell nucleus. All samples were rinsed with PBS to remove residual waste and submerged in 1× PBS during imaging. All reagents were purchased from Beyotime (China) unless further specified. Analysis of cell pseudopodia, volume, and sphericity was based on single cells not in contact with the other cells. Optical image stacks of single cells were obtained with a 0.5-μm *z*-axis interval. Imaris software was used to calculate the cell pseudopodia, volume, and sphericity. The threshold for 3D visualization was held constant for each batch of experiments. Fibriltool, an ImageJ plug-in, was used to quantify the organization of actin organization and polarization (*72*).

For evaluating the maturity of focal adhesion, hydrogel constructs were first fixed with 4% paraformaldehyde for 24 hours and permeabilized with 0.5% Triton X-100 in 1× tris-buffered saline (TBS) for 5 min. Samples were retrieved for antigen using proteinase K (20 μg/ml) retrieval buffer (pH 8.0) before blocking with 10% goat serum at 37°C for 2 hours and then incubated with mouse monoclonal vinculin antibody in 1% bovine serum albumin (BSA) at 4°C overnight. Next, the constructs were treated with secondary antibody and tetramethyl rhodamine isothiocyanate (TRITC)–conjugated phalloidin at room temperature (RT) in the dark for 1 hour. Last, the hydrogel constructs were incubated with DAPI in TBS at RT for 1 min. The following antibodies/reagents were used for immunostaining: actin cytoskeleton/focal adhesion staining kit (Sigma-Aldrich, USA, FAK100) and fluorescein isothiocyanate (FITC)–conjugated goat anti-mouse immunoglobulin G (IgG) (H+L) (Proteintech, China, SA00003-1). All reagents were purchased from Beyotime (China) unless further specified. The intracellular localization and fluorescence intensity of vinculin were analyzed using Imaris spot analysis software.

### Histological assessment

Hydrogel constructs were first fixed with 4% paraformaldehyde, dehydrated in a graded series of sucrose, embedded in optimal cutting temperature compound (OCT) (Leica, Germany), frozen, and sectioned. SO staining was carried out to identify proteoglycans in hydrogel constructs. For assessing the level of cell death in each of the hydrogels, TUNEL (Beyotime, China, C1086) staining was conducted by visualizing a marker of late apoptosis according to the manufacturer’s instruction. Samples were permeabilized with 0.5% Triton X-100 in 1× TBS for 5 min and then retrieved for antigen using proteinase K (20 μg/ml) retrieval buffer (pH 8.0), followed by incubation with TUNEL detection of liquid at RT in the dark for 1 hour. Last, the hydrogel constructs were incubated with DAPI in TBS at RT for 1 min. The numbers of TUNEL^+^ cells were automatically quantified on high-resolution images using ImageJ software.

For evaluating the type II collagen deposition, hydrogel constructs were first fixed with 4% paraformaldehyde for 24 hours and permeabilized with 0.5% Triton X-100 in 1× TBS for 5 min. Samples were retrieved for antigen using proteinase K (20 μg/ml) retrieval buffer (pH 8.0) before blocking with 10% goat serum at 37°C for 2 hours and then incubated with mouse monoclonal type II collagen antibody in 1% BSA at 4°C overnight. Next, the constructs were treated with secondary antibody and TRITC-conjugated phalloidin at RT in the dark for 1 hour. Last, the hydrogel constructs were incubated with DAPI in TBS at RT for 1 min. The following antibodies/reagents were used for immunostaining: type II collagen (NOVUS, USA, NB600-844) and FITC-conjugated goat anti-mouse IgG (H+L) (Proteintech, China, SA00003-1). All reagents were purchased from Beyotime (China) unless further specified. The fluorescence intensity of type II collagen was analyzed using Imaris spot analysis software.

### Western blot

Proteins were isolated from MSCs in hydrogels using 200 μl of radioimmunoprecipitation assay lysis buffer (Beyotime, China) with protease inhibitor cocktail (Cell Signaling Technology, USA) and phosphatase inhibitor cocktail (Beyotime, China) per gel. Proteins were separated by electrophoresis in Stain-Free FastCast gels (Bio-Rad, USA) and transferred to Sequi-Blot polyvinylidene difluoride membrane (Bio-Rad, USA) by using the Trans-Blot Turbo Blotting System (Bio-Rad, USA). Membranes were probed for p-MLC, MLC, Sox-9, ROCK1-cleaved, ROCK1, or β-actin as a loading control (ABclonal, China). Horseradish peroxidase–conjugated goat anti-mouse or goat anti-rabbit secondary antibodies (ABclonal, China) were then applied and incubated for 1 hour at RT. Signals were displayed with a chemiluminescent ECL (electrochemiluminescence) substrate (Beyotime, China) under an imaging system (Bio-Rad, USA).

### Gene expression analysis

RNA in each of the cell-hydrogel constructs was harvested with the RNeasy Mini Kit (QIAGEN, USA) and then reverse-transcribed into complementary DNA (cDNA) using the iScript cDNA Synthesis Kit (Bio-Rad, USA). Quantitative real-time polymerase chain reaction (PCR) was performed using the CFX96 real-time PCR detection system (Bio-Rad, USA) with Sso-Fast EvaGreen Supermix (Bio-Rad). Relative levels of each targeted gene were calculated by the ΔΔCt method (*73*). The chondrogenic markers (including *SOX-9*, *ACAN*, and *Col2a1*) were quantified and internally normalized to the housekeeping gene glyceraldehyde-3-phosphate dehydrogenase (*GAPDH*). The sequences of the primers are given in Table 1.

**Table 1.** Sequences of primers for RT-PCR.

Gene	Forward primer	Reverse primer
*GAPDH*	TCGGAGTGAACGGATTTGGC	TTCCCGTTCTCAGCCTTGAC
*SOX-9*	AAGCTCTGGAGACTGCTGAA	CCCATTCTTCACCGACTTCCT
*ACAN*	GGCCACTGTTACCGTCACTT	GTCCTGAGCGTTGTTGTTGAC
*Col2a1*	TCTGTGAAGACACCAAGGACTG	TTCTCCTTTCTGCCCCTTTGGT

### Biochemical analysis

Biochemical analysis was conducted as described previously (*20*). In brief, hydrogel constructs were removed from induction medium and freeze-dried. Then, the gels were digested with papain solution (100 μg/ml; Sigma-Aldrich, USA) in 0.2 M Na_2_-HPO_4_-NaH_2_PO_4_, sodium acetate (8 mg/ml), EDTA disodium salt (4 mg/ml), and cysteine hydrochloride buffer (0.8 mg/ml) at 65°C for 12 hours. sGAG contents were quantified with the Blyscan sGAG assay kit (Biocolor, Newtownabbey, UK). The fluorescent dye-based DNA Quantification Kit (PicoGreen, Invitrogen, USA) was used to measure the amount of DNA in hydrogels according to the manufacturer’s protocol.

### Inhibition of Rho/ROCK signaling

For pharmacological inhibition studies, the inhibitors were added to the induction medium following 1 day of culture. The concentrations used for each inhibitor were 10 μM for Y-27632 (Beyotime, China) and 5 μM for Blebbistatin (Beyotime, China). These concentrations matched those used in similar studies (*74*, *75*).

### Statistical analysis

Statistical comparisons were performed with a two-tailed Student *t* test when only two groups were being compared, and one- or two-way analysis of variance (ANOVA) with Tukey’s multiple comparisons test was used to make pairwise comparisons between multiple groups by GraphPad Prism 7.0 statistical software (GraphPad Software). In particular, the statistical comparisons for length and width of collagen fibers were performed with one-way ANOVA with Kruskal-Wallis test to make pairwise comparisons between multiple groups. Statistical significance was set to *P* < 0.05.
