# Supplementary material for: Collagen hydrogel viscoelasticity regulates MSC chondrogenesis in a ROCK-dependent manner
Source: Sci Adv. 2023 Feb 10;9(6):eade9497. doi: 10.1126/sciadv.ade9497 (PMC9916999; doi:10.1126/sciadv.ade9497)
Supplement: Supplementary file 1 — Figs. S1 to S9 [file sciadv.ade9497_sm.pdf]

Science Advances
AAAS

Supplementary Materials for

Collagen hydrogel viscoelasticity regulates MSC chondrogenesis in a ROCK-dependent manner

Danyang Huang *et al.*

Corresponding author: Yun Xiao, yun.xiao@scu.edu.cn

Sci. Adv. **9**, eade9497 (2023)
DOI: 10.1126/sciadv.ade9497

This PDF file includes:

Figs. S1 to S9

Supplementary Materials

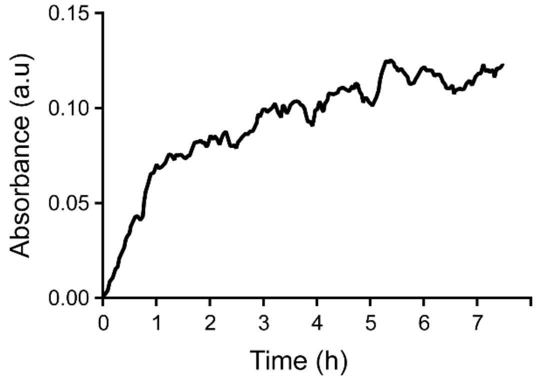

Fig S1. Turbidity curve of collagen solution obtained at 313 nm and 4 °C. Collagen concentration: 3 mg/ml.

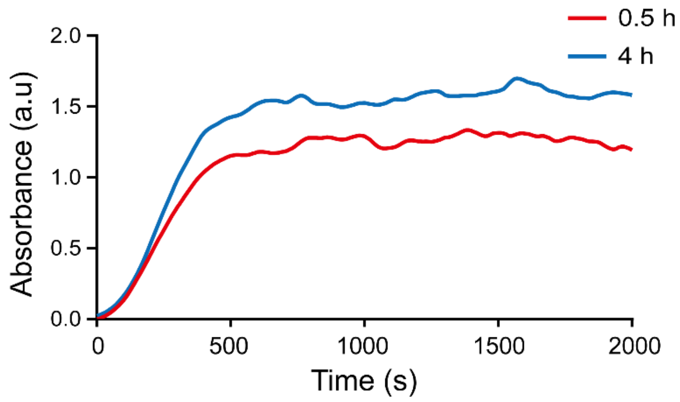

Fig S2. Turbidity curves of collagen solution obtained at 313 nm and 37 °C after low-temperature incubation for 0.5 and 4 h. Collagen concentration: 3 mg/ml.

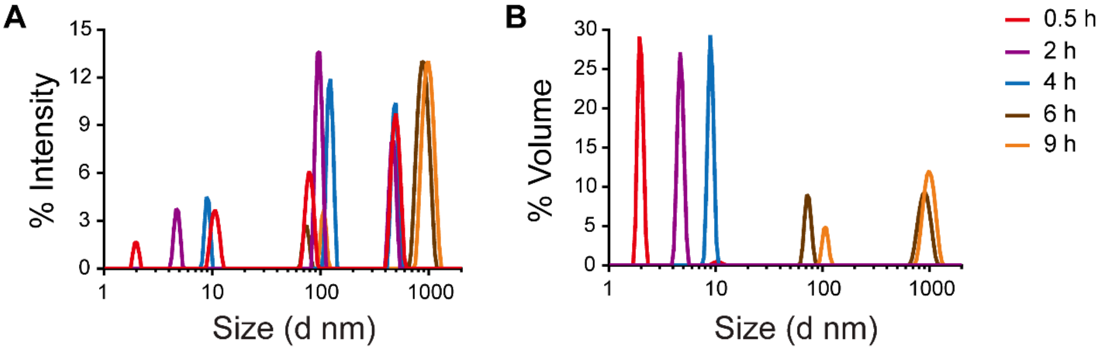

Fig S3. The size distribution of collagen fibers in collagen solutions with different low-temperature incubation time. (A) Light intensity particle size distribution of collagen fibers. **(B)** Volume particle size distribution of collagen fibers. Collagen concentration: 3 mg/ml.

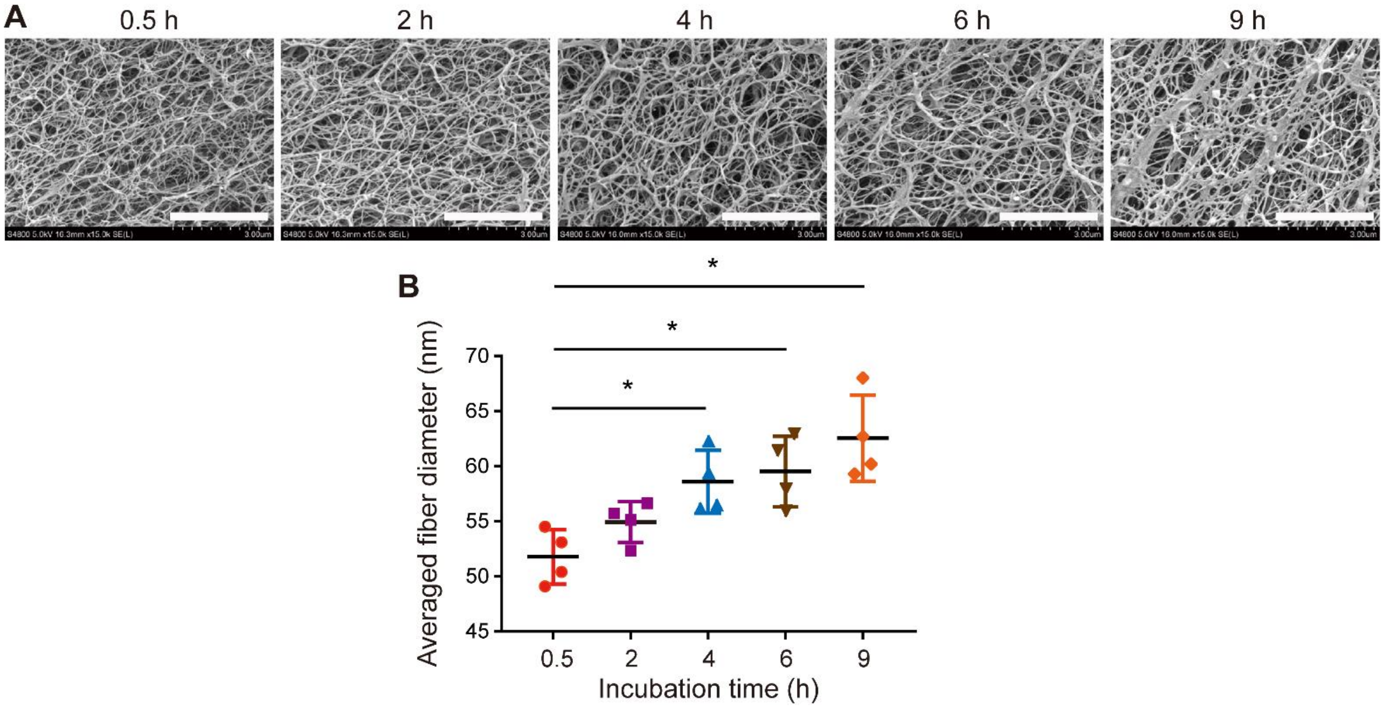

Fig S4. SEM characterization of collagen fibers with different low-temperature incubation times. (A) SEM images of critical point-dried collagen hydrogels with various low-temperature incubation time. Scale bars, 3 µm. **(B)** Quantification of the diameter of collagen fibers (averaged from four regions; *$P < 0.05$). Collagen concentration: 3 mg/ml.

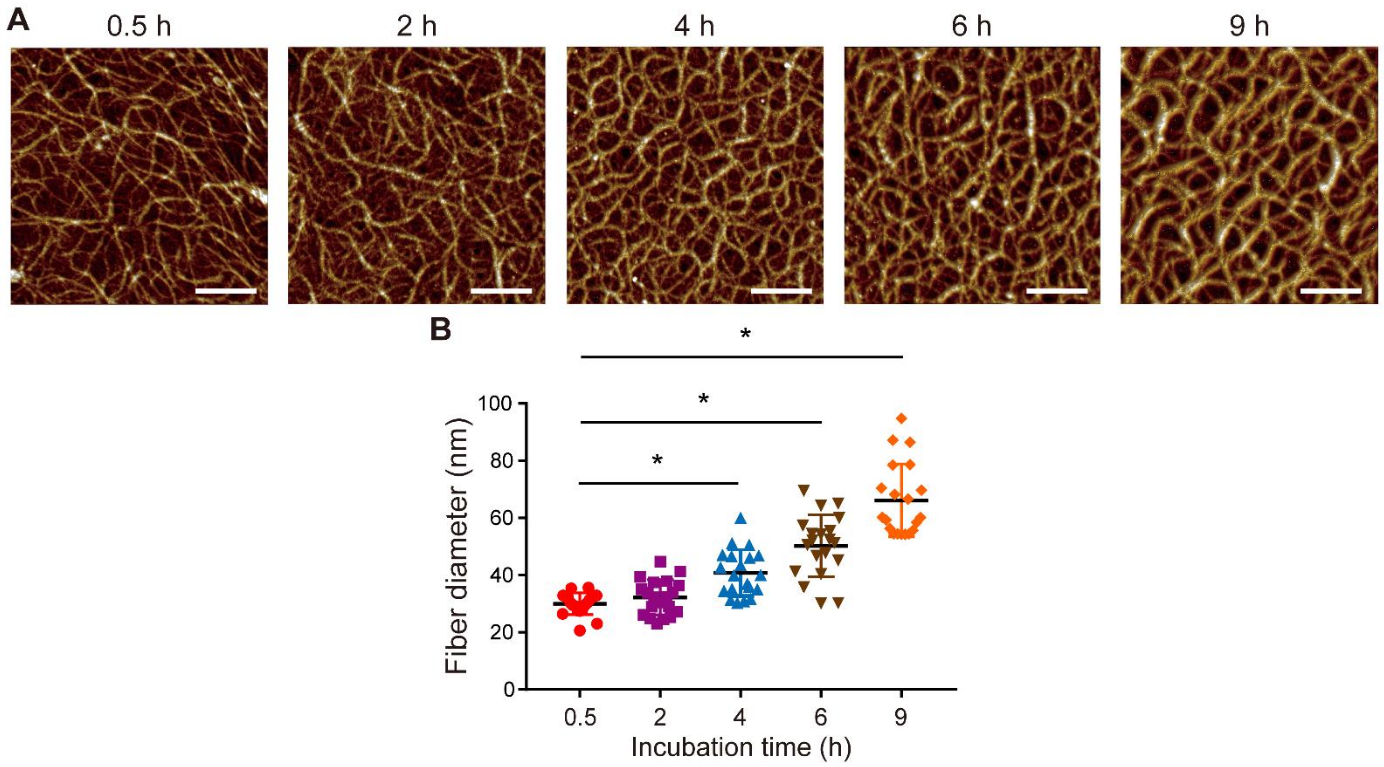

Fig S5. AFM characterization of collagen fibers with different low-temperature incubation times. (A) AFM images of dried collagen hydrogels with various low-temperature incubation time. Scale bars, 500 nm. **(B)** Diameter of individual collagen fibers with various low-temperature incubation time (n = 21 single fibers from three regions per each condition; *$P < 0.05$). Collagen concentration: 3 mg/ml.

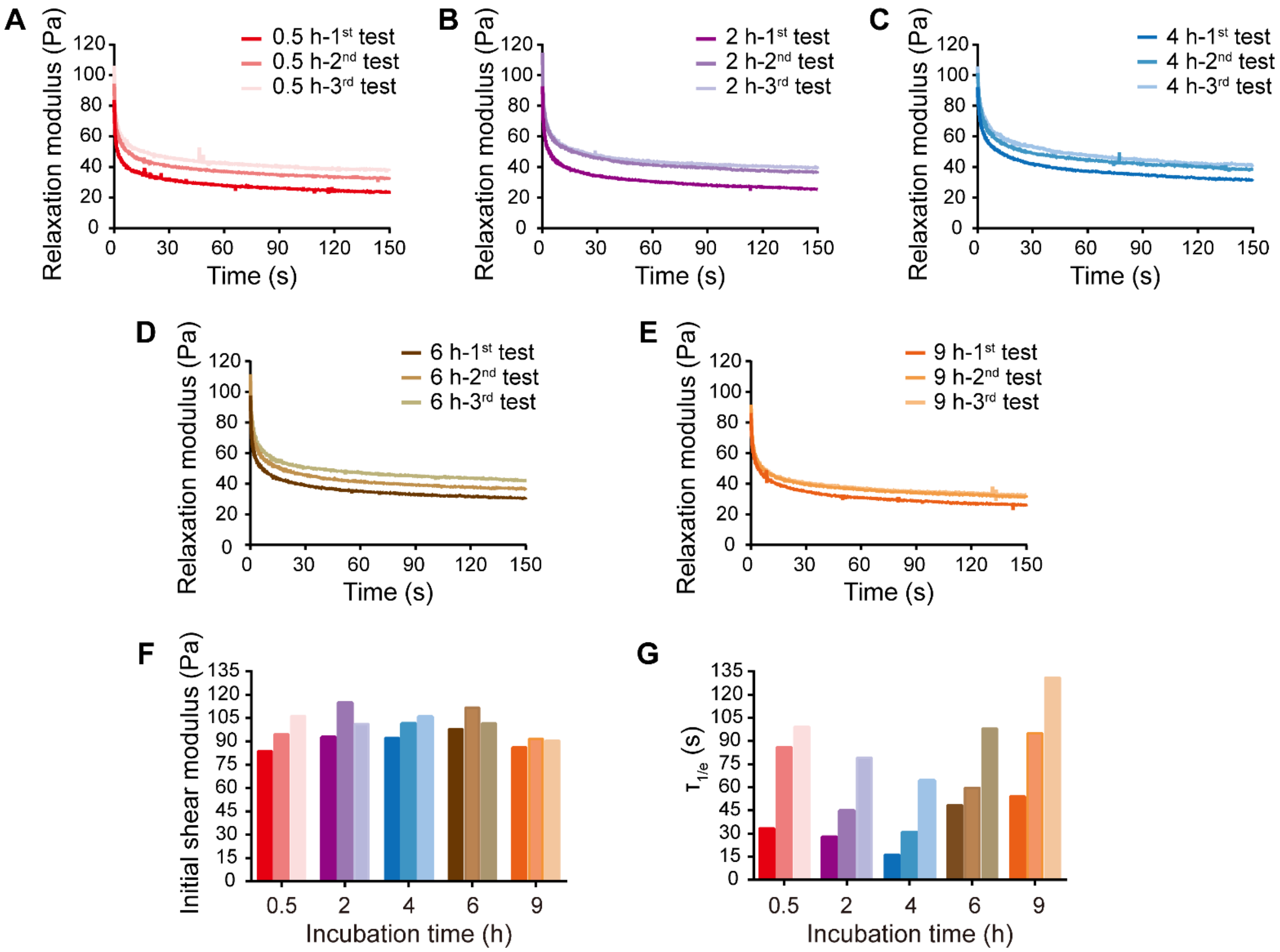

Fig S6. Repeated stress relaxation tests of collagen hydrogels with various low-temperature incubation times. (A-E) Representative repeated stress relaxation curves of collagen hydrogels with various low-temperature incubation time at a strain of 10%. **(F)** Initial shear modulus as determined by repeated stress relaxation tests of collagen hydrogels. **(G)** Time scale of repeated stress relaxation, $\tau_{1/e}$, for the different collagen hydrogels.

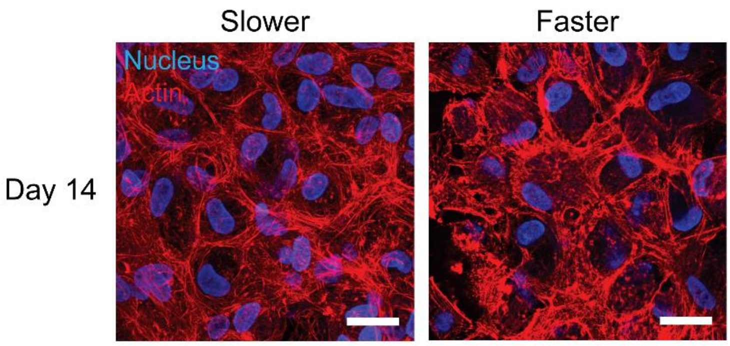

Fig S7. Representative images of fluorescence staining for F-actin of MSCs cultured in hydrogels for 14 days. Scale bars, 10 µm.

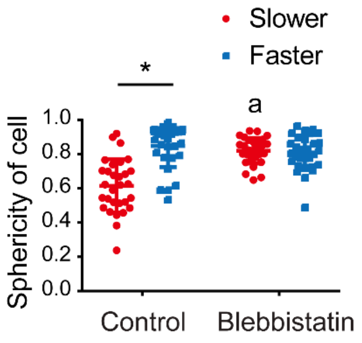

Fig S8. Quantification of sphericity of cells in the presence of blebbistatin or Y-27632 (n = 30 single cells from three biological replications per each condition; *$P < 0.05$); a indicates a significant difference (*$P < 0.05$) compared with the slower hydrogels in control group.

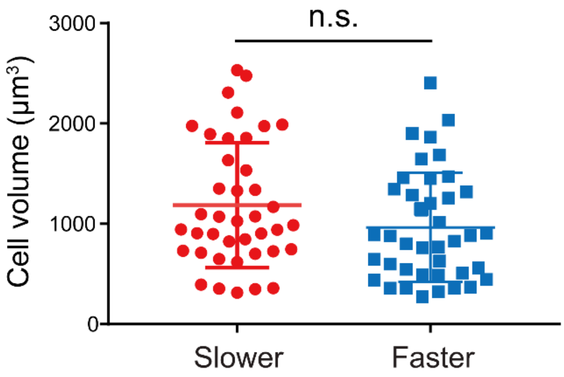

Fig S9. Quantification of cell volume in slower/faster-relaxing groups on day 7 (n = 30 single cells from three biological replications per each condition; $P = 0.4073$).
